# The Role of Type 2 Inflammation in *Schistosoma*-Induced Pulmonary Hypertension

**DOI:** 10.3389/fimmu.2019.00027

**Published:** 2019-01-24

**Authors:** Claudia S. Mickael, Brian B. Graham

**Affiliations:** Program in Translational Lung Research, Department of Medicine, University of Colorado Anschutz Medical Campus, Aurora, CO, United States

**Keywords:** schistosomiasis, pulmonary hypertension, Th2 inflammation, transforming growth factor β (TGF-β), vascular remodeling

## Abstract

Approximately 5% of individuals chronically infected with *Schistosoma mansoni* develop pulmonary hypertension (PH). The disease is progressive and often fatal, and treatment options are palliative, not curative. Recent studies have unraveled major players of the Th2 inflammation axis in the *Schistosoma*-induced PH pathology using murine models and studying human samples. TGF-β signaling is a link between the Type 2 inflammation and vascular remodeling, and specifically Thrombospondin-1 (TSP-1) is upregulated by the inflammation and activates TGF-β. Overall, the current model for the pathogenesis of *Schistosoma*-induced PH is that deposition of *Schistosoma mansoni* eggs in the pulmonary vasculature results in localized Th2 inflammation, leading to TGF-β activation by TSP-1, and the active TGF-β then results in vascular remodeling and PH.

## Introduction

Schistosomiasis is caused by infection with parasites from the genera *Schistosoma*, affecting around 200 million people worldwide. There are several species that infects humans, but *Schistosoma japonicum, hematobium*, and *mansoni* are the most prevalent. Although there are effective anti-helminthic drugs available, schistosomiasis is considered a neglected tropical disease, since many social and economic factors impede access to effective prevention and treatment (1, 2).

Pulmonary hypertension (PH) is an obstructive disease of the lung vasculature, defined as a mean pulmonary artery pressure ≥ 25 mmHg (a normal pulmonary artery pressure is usually 12–18 mmHg). There are 5 categories of PH designated by World Health Organization (WHO) consensus guidelines (3). The first category, also called WHO Group 1, involves the pulmonary arterial system specifically, and is more precisely termed pulmonary arterial hypertension (PAH). There are several etiologies which result in WHO Group 1 PAH, including schistosomiasis (3).

Around 10% of chronically and recurrently infected patients will develop the severe hepatosplenic form of the disease which manifests as pre-portal fibrosis, also known as “pipestem fibrosis.” Most of the *Schistosoma*-associated PAH cases develop in conjunction with hepatic portal fibrosis. About 5 to 8% of the patients with severe schistosomal liver disease will subsequently develop pulmonary hypertension (PH), for an overall incidence of approximately 0.5% of those with acute schistosomiasis developing *Schistosoma*-PAH, or about 1 million worldwide (3–5).

The physiology of portal hypertension leads to opening of portocaval shunts and embolization of *Schistosoma* eggs from the portal venous system to the systemic venous system. These shunts facilitate the embolization of eggs which travel through the right heart and lodge in the pulmonary vasculature. *Schistosoma* eggs have a short axis diameter of about 50 μm, resulting in the eggs ending up in small, pre-capillary vessels of this internal diameter. There the eggs trigger a robust immune response, characterized by Type 2 immunity including eosinophils, macrophages, lymphocytes (including Th2 CD4 T cells) and fibrocytes, with strong expression of the cytokines IL-4, IL-5, IL-10, and IL-13 (6).

Factors which modulate the burden of eggs delivered to the lung can modify the severity of associated PH. For example, blockade of the IL-10 receptor in a mouse model of schistosomiasis led to portal hypertension and increased accumulation of eggs in the lungs, resulting in more severe pulmonary disease (7). In another report, heterozygosity of BMPR2 (a mutation which can separately result in heritable PAH) resulted in worse portal hypertension and increased shunting of eggs to the lungs, similarly worsening the PH phenotype (8).

## Type 2 Immune Response

Initially it was thought, based on histopathologic analysis of autopsy specimens from individuals that died of schistosomiasis-associated PAH, that the PH resulted solely from direct, mechanical obstruction of the pulmonary vasculature by diffuse egg embolization. As a consequence, schistosomiasis was initially categorized in the WHO Group 5 classification system of PH, within the “miscellaneous/multifactorial” group. However, at the 2008 world PH consensus conference, schistosomiasis was moved to WHO Group 1 PAH, based on several observations including:

- The histopathology of schistosomiasis-associated PAH resembles other forms of PAH, including characteristic plexiform lesions;
- Patients with schistosomiasis-associated PAH who received late treatment with the antihelminthic praziquantel still died of the condition, with significant pulmonary vascular disease but now with the absence of eggs in the lung; and
- Patients with schistosomiasis-associated PAH clinically respond to pulmonary artery vasodilators, such as sildenafil, as do patients with other forms of WHO Group 1 PAH.

**Figure 1:**
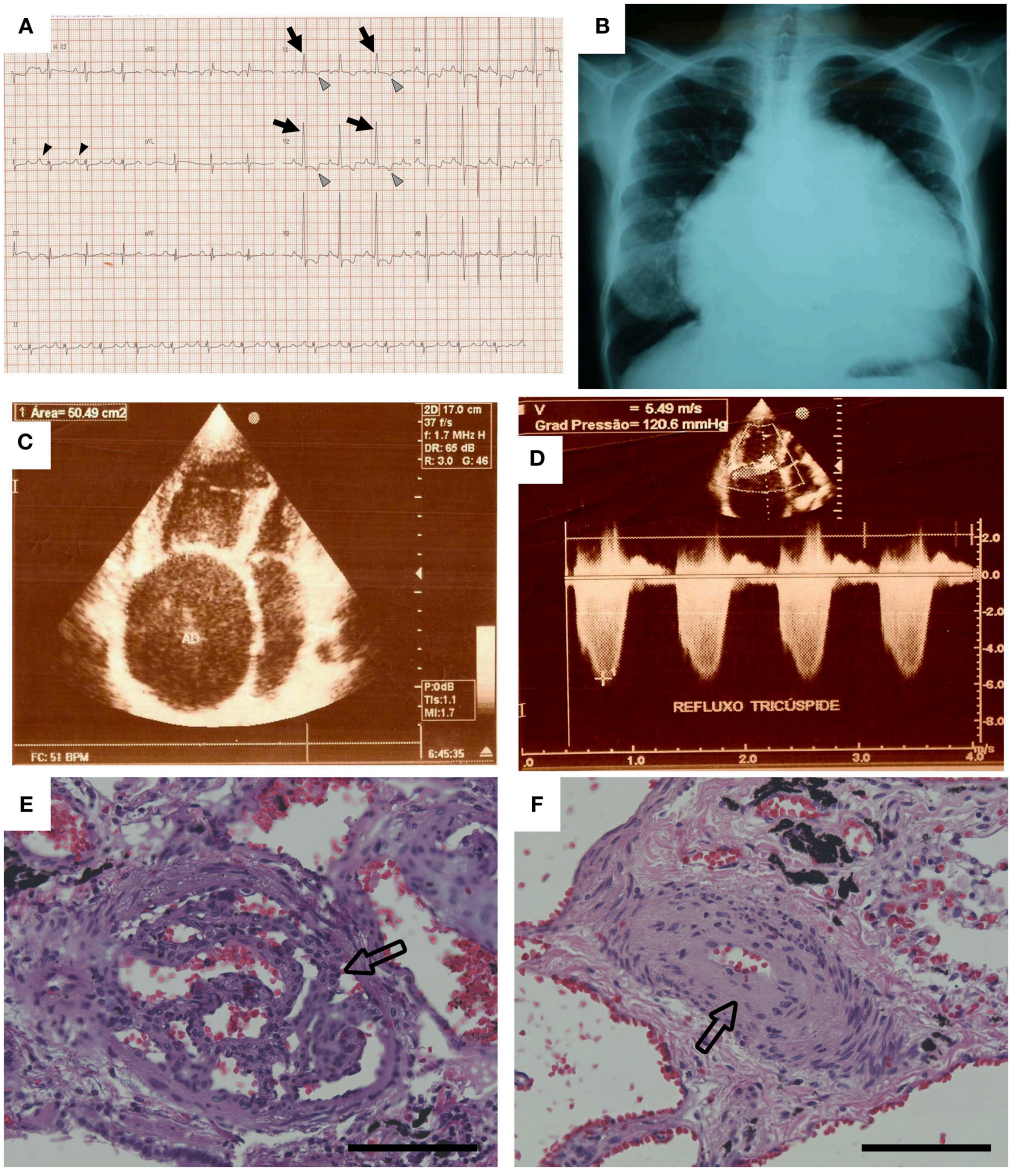
Data from the case example. **(A)** The patient's electrocardiogram showed evidence of right atrial enlargement (black arrowheads) and right ventricular hypertrophy. Arrows show large R waves, and gray arrowheads show inverted T waves in V1 and V2. **(B)** The chest radiograph showed severe cardiomegaly. **(C,D)** Echocardiography revealed severe enlargement of the right atrium and right ventricle, and Doppler examination of the tricuspid valve resulted in an estimated pressure gradient of 120 mm Hg. Pulmonary pathology found on autopsy included **(E)** plexiform lesions and **(F)** concentric intimal thickening. Hematoxylin and eosin stain, original magnification × 20. Scale bars are 100 μm [Reprinted with permission from Graham et al. (11)].

Schistosomiasis-associated PAH is likely proximately triggered by the immune response to the eggs in the lung vasculature. However, once the inflammation has been ongoing long enough and to an adequate degree, permanent schistosomiasis-associated PAH is established, and anti-helminthic drugs are no longer effective (9, 10). At this point, the treatment options are limited to vasodilator therapy, which is palliative, not curative. Patients eventually die of right ventricle failure resulting from the vascular disease, which histopathologically presents with a combination of media thickening and intima remodeling (11) (Figure 1).

Chronic hepatic disease can result in porto-pulmonary hypertension, likely due to inadequate clearance of toxins which results in pulmonary vascular injury, coupled with increased shear stress in the setting of elevated cardiac output with liver disease. Thus porto-pulmonary hypertension from preportal fibrosis is likely an additional contributor to human schistosomal PAH.

**Figure 2:**
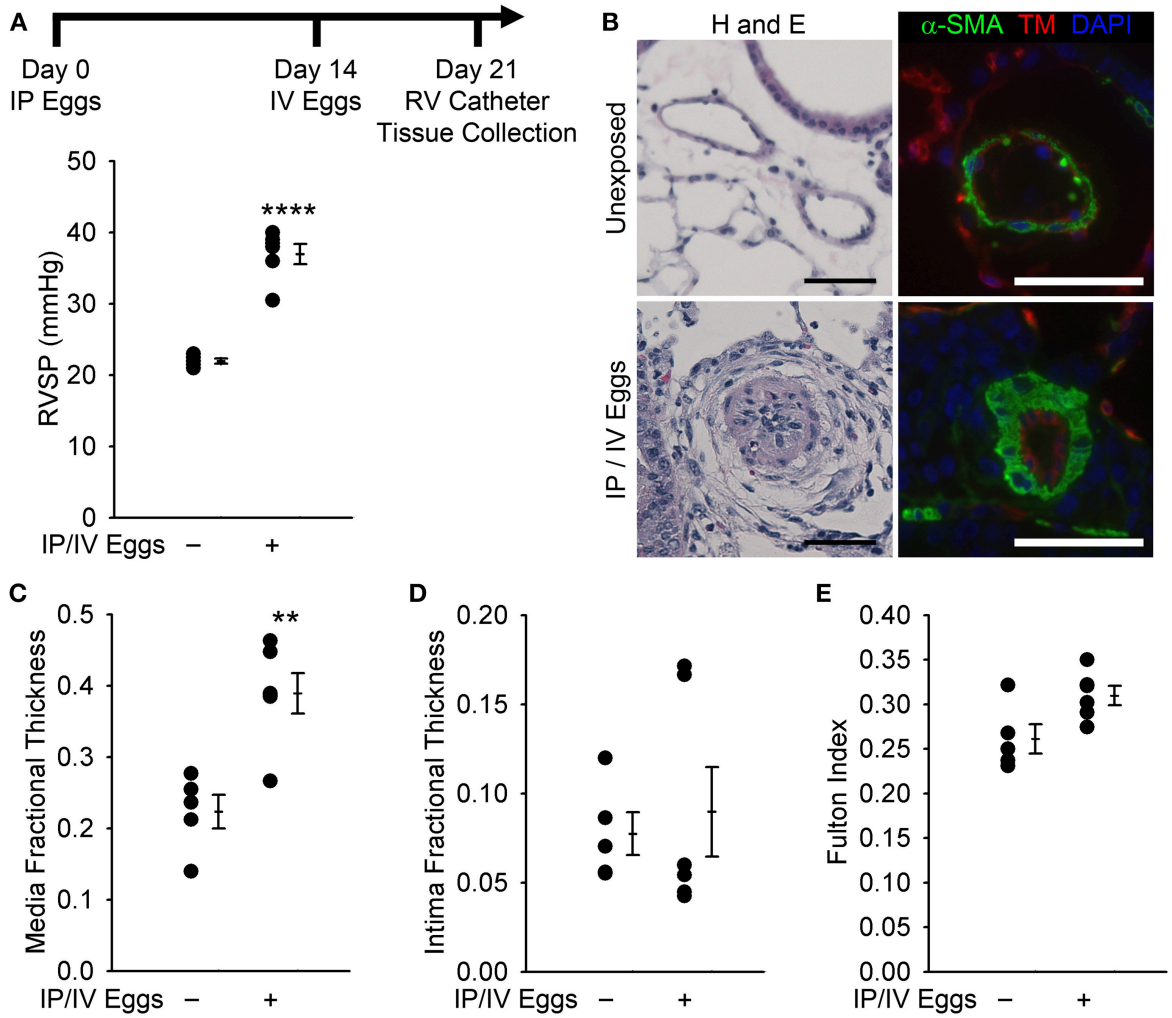
Mice exposed to *Schistosoma mansoni* eggs develop pulmonary hypertension (PH) and vascular remodeling. **(A)** Mice intraperitoneally sensitized to *S mansoni* eggs followed by intravenous augmentation have an increase in right ventricular (RV) systolic pressure (RVSP; mean ± SE; *n* = 5–6 mice per group; rank-sum test, ^****^P < 0.001; this experiment was repeated 3 times with similar results). **(B)** Representative hematoxylin and eosin (H&E) and immunofluorescence staining for α-smooth muscle actin (α-SMA) and thrombomodulin of unexposed and intraperitoneal/ intravenous egg–exposed mouse lungs (scale bars, 50 μm). **(C,D)** Quantitative fractional thickness of the pulmonary vascular media and intima in unexposed and intraperitoneal/ intravenous egg–exposed mice (mean ± SE; *n* = 5–6 mice per group; rank-sum test, ^**^P < 0.01; *P* = 0.66 for intima thickness). **(E)** Fulton index [RV/(LV+S)] of unexposed and intraperitoneal/intravenous egg– exposed mice (mean ± SE; *n* = 5–6 mice per group; rank-sum test, *P* = 0.052) [Reprinted with permission from Graham et al. 12].

In order to unveil the mechanisms by which the host inflammatory response to the *Schistosoma* parasite results in pulmonary vascular disease, our lab developed a simplified mouse model of the condition, which adopted the commonly used egg sensitization-egg challenge model used in immunology to study Type 2 lung granulomas. In the PH model, mice are intraperitoneally (IP) sensitized with *Schistosoma mansoni* eggs, followed by challenge with intravenous (IV) injection by tail vein of purified and live eggs 2 weeks thereafter. The IP sensitization is performed with either frozen or live eggs: we have not detected a different phenotype between the two. One week later, the mice undergo catheterization of the right ventricle, to measure the right ventricle systolic pressure (RVSP), and tissue collection is performed following the catheterization procedure (12). This model is very robust and reproducible, as demonstrated by a significantly higher RVSP as well as pulmonary vascular media thickening in wild type challenged mice as compared to unchallenged animals (Figure 2). The fact that IP/IV eggs alone are able to trigger substantial inflammation PH in mice suggests that in humans the egg embolization to the lungs is likely a major driver of the PAH, which may be further compounded by liver disease. Furthermore, IP sensitization is a necessary prerequisite, as IV eggs alone do not induce PH, supporting the concept that a robust immune response is required for the development of PH (13).

Other laboratories have used cercariae infection of mice, which also results in a PH phenotype. However, the cercariae model is more heterogenous, with the PH severity showing significant variation between mice and specifically correlating with the degree of egg embolization into the lungs which varies substantially from mouse to mouse even in inbred strains (8, 14). The embolic egg burden can be titrated somewhat based on the number of cercariae used, but there is a tradeoff between enough eggs to induce PH versus severe enough liver disease resulting in premature death of the experimental animal. Another potential limitation of the cercariae model is that the model includes liver disease which can separately cause PH, potentially confounding interpretation of drivers of the PH phenotype. For example, carbon tetracholoride-induced cirrhosis can also induce PH in mice (15). The cercariae infection model can be modified to result in a more robust and reproducible PH phenotype by administering a bolus of eggs to the lungs by tail vein IV administration, on top of the chronic cercariae infection, although we see unclear benefit of this model vs. the IP sensitization/IV challenge model (13).

Both the IP/IV and cercariae infection models are substantially limited as they do not capture the persistence of disease. As noted, humans with Schistosoma-PAH typically following decades of chronic and recurrent infection are not significantly benefited by treatment with praziquantel to eradicate the parasite. In the IP/IV model, the single bolus of eggs into the lungs results in transient inflammation, lasting 2–3 weeks, which self-resolves as the eggs are degraded by the immune system and the antigenic trigger is cleared. In the cercariae infection model, despite a exposure duration that lasts several months, treatment with praziquantel similarly causes reversal of the PH phenotype (16).

However, we and others believe that the schistosomiasis exposure model, by either IP/IV eggs or cercariae infection, can be useful to understand the pathogenesis of the disease, specifically linking the clear antigenic trigger with the vascular remodeling phenotype. Using the IP sensitization/IV challenge model, our lab has dissected a series of mechanistic steps by which the Type 2 immune response results in pulmonary vascular remodeling.

We have observed that *Schistosoma* exposed mouse lung tissue contains higher levels of IL-4 and IL-13, while mice with blocked Type 2 immunity by combined deficiency of IL-4 and IL-13 were protected from *Schistosoma*-PH, with a significant reduction in right ventricle systolic pressure, less pulmonary vascular media thickening, and smaller peri-egg granuloma volumes compared with wild type mice (17). This protection was not observed in mice singly deficient for either IL-4 or IL-13, probably due to redundant effects of the other ligand for their shared receptor IL-4Rα/IL-13Rα1. This data was corroborated by immunostaining of human lung tissue of patients with *Schistosoma*-induced PAH, which presented higher staining intensity for IL-13, IL-4Rα, phospho-signal transducer and activator of transcription factor 6 (pSTAT-6, a key target of IL-4 and IL-13), and periostin (another target of IL-4 and IL-13 signaling), which reinforces the clinical importance of these findings.

We sought to determine the origin of IL-4 and IL-13 using IL-4^GFP^ and IL-13^YFP^ reporter mice, and observed that about 75% of each derived from CD3+CD4+ T cells (17). Of the CD4 T cells present, about 3.7 and 1.1% were positive for IL-4 and IL-13, respectively. We confirmed the bone marrow origin of these IL-4 and IL-13 producing cells by transferring *Il4*^−/−^*Il13*^−/−^ BM cells into lethally irradiated wild type-mice recipients, and observed protection from *Schistosoma* induced PH as well as lower levels of IL-4 and IL-13 in the lungs when compared to wild-type mice that received wild-type BM donor cells. Of note, an increased density of CD4 T cells has also been reported in the lung tissue of patients who died of schistosomiasis-associated PAH based on analysis of tissues collected at autopsy (18).

Other cells like eosinophils, basophils, mast cells and ILC2 cells can also secrete IL-4 and IL-13 (19, 20), and could also contribute to the *Schistosoma*-PH phenotype. It has been previously reported that eosinophils and basophils are both dispensable in the liver phenotype resulting from *Schistosoma* infection (21, 22), so we suspect these cells may well be dispensable for the PH phenotype as well, but this has not been tested experimentally. Overall, we suspect that Th2 CD4^+^ T cells in particular are critical for the Type 2 immunity which drives the development of *Schistosoma*-PH in mice and possibly humans as well.

There may be a direct effect of IL-13 and/or IL-4 on the lung vascular cells which could result in PH. *In vitro* studies have demonstrated that treatment of human pulmonary artery endothelial cells (HPAECs) with IL-13 upregulates Rictor expression via reduction of miR-424/504, enhancing HPAEC migration which is associated with PH (23). IL-13 can also stimulate pulmonary artery smooth muscle cell migration *in vitro* (14). However, others have shown IL-13 can suppress pulmonary artery smooth muscle cell proliferation (24).

## TGF-β Activation by Thrombospondin-1 (TSP-1)

Based on our studies, we consider TGF-β to be the critical downstream signaling mediator which links proximate Type 2 inflammation to distal vascular remodeling. Dysregulated TGF-β signaling has also been implicated in many different forms of WHO Group 1 PAH, including heritable disease (resulting from mutations in the TGF-β signaling family, mostly commonly BMPR2), idiopathic, and auto-immune disease triggered PAH. TGF-β has the ability to induce pathology in pulmonary artery endothelial and smooth muscle cells including promoting vasoconstriction, hypertrophy, proliferation and apoptosis—phenotypes which result in vascular lumen obstruction (12, 25, 26).

We have previously observed that TGF-β signaling is increased in the pulmonary vessels in patients with *Schistosoma* associated PAH and in *Schistosoma* challenged mice (12, 13). Another group has reported higher levels of TGF-β1 in serum samples of patients with *Schistosoma* associated PAH, compared to patients with acute *Schistosoma* infection, corroborating these results (27). TGF-β isoform 1 (but not isoforms 2 or 3) is upregulated at the mRNA level in *Schistosoma*-PH mice, so we suspect but have not yet directly tested that this isoform in particular drives the pathology. We also found that expression of the canonical TGF-β target phospho-Smad2/3, which is upregulated in the pulmonary vasculature following *Schistosoma* exposure (12, 13), was reduced in double-deficient *Il4*^−/−^*Il13*^−/−^ mice compared to wild-type mice (12), supporting that this is a target of Type 2 immunity. We have found that blocking TGF-β signaling by the pan-neutralizing antibody 1D11, by TGF-β receptor small molecule inhibitors, or by deficiency of the intracellular canonical signaling mediator Smad3 all protected mice from *Schistosoma*-induced PAH, exemplified by lower RVSP as well as reduced media thickness when compared to mice treated with the appropriate controls (12).

As noted above, another group using the cercariae model of *Schistosoma*-PH found that mice with heterozygosity of the TGF-β family receptor BMPR2 have a worsened PH phenotype, primarily mediated by increased liver disease and more shunting of the parasite eggs to the lungs (8): dominant negative BMPR2 mutations are a common cause of heritable human PAH.

It has been previously described that TGF-β is highly regulated at the level of activation, as it is secreted into the extracellular matrix in an inactive form, bound to the latency-associated peptide (LAP). Several compounds, including serine proteases, integrins and thrombospondins (TSPs) activate TGF-β by removing the active ligand from the LAP complex. We assessed TSP-1 levels in the lung and we observed increased mRNA levels of *Thbs1* (the TSP-1 encoding gene) in *Schistosoma-*exposed wild type mice compared to unexposed animals. Similar to the TGF-β signaling phenotype above, we observed a significant decrease in protein and mRNA levels of TSP-1 in the double deficient *Il4*^−/−^*Il13*^−/−^ mice, compared to wild type mice, indicating that TSP-1 expression is Th2 dependent (28).

The TSP-1 protein contains a conserved amino acid motif (KRFK) that interacts with a corresponding LSKL amino acid sequence present in the LAP, resulting in mechanical alteration of the LAP structure and the release of active TGF-β (29). We competitively blocked the TGF-β-activating function of TSP-1 by treating *Schistosoma*-challenged and unchallenged mice with a synthetic LSKL peptide, as compared to scrambled control peptide SLLK, and observed the mice treated in this manner were protected from PH, exemplified by lower RVSP and media thickness. In addition, we observed that TSP-1 blockade did not change IL-4 and IL-13 levels at protein or mRNA levels, confirming that this event occurs downstream of both cytokines (28).

We then performed flow cytometry of dispersed lung murine cells and observed the emergence of a TSP-1^+^ population in the *Schistosoma*-exposed group, which could be subdivided into two subgroups: one was CD64^lo^, MerTk^int^ and Ly6C^hi^ which is consistent with intravascular Ly6C+ monocytes, and another population characterized by CD64^int^, MerTk^hi^ and Ly6C^int^ which resembles the intra-parenchymal macrophage population. Most of the cells in these groups were TSP-1 positive, and were also likely to express *Tsp1* mRNA, since the TSP-1 signal was intracellular. In particular, we hypothesized that the interstitial macrophage population was derived or recruited from the circulating Ly6C monocyte population. To investigate if bone marrow (BM) derived cells (including Ly6C+ monocytes) are critical, we transplanted BM cells from TSP-1^−/−^ mice into lethally-irradiated wild-type mice, followed by *Schistosoma* challenge. We observed that these mice were significantly protected from *Schistosoma*-PH, with lower RVSP levels and less media thickening compared to mice that received wildtype BM cells (28).

Ly6C+ monocytes express CCR2, and are recruited into tissues by ligands such as CCL2, CCL7, and CCL12 which bind to CCR2. By flow cytometry, we found that the lung interstitial macrophages from *Schistosoma*-exposed mice expressed higher levels of *Ccl2, Ccl7*, and *Ccl12*, suggesting that these macrophages, activated by the proximate Th2 CD4 T cells, call in the Ly6C^+^ monocytes. Next we blocked the recruitment of Ly6C^+^ monocytes by BM transplant of cells derived from *Ccr2*^−/−^ mice into lethally irradiated wild-type animals. We observed these mice when challenged with *Schistosoma* had significantly lower RVSP and less right ventricle hypertrophy compared to animals that received control wild-type BM cells. We also analyzed lung dispersed cells by flow cytometry and observed that challenged animals that had received *Ccr2*^−/−^ BM had increased numbers of intravascular monocytes, similar to the phenotype of wild-type mice, but a failure of the monocytes to enter into the lung parenchyma (28).

To address if the TSP-1 pathological function correlates with TGF-β activation, we measured the concentration of active TGF-β levels in murine lung lysates using a cell line transfected with a *Pai1* promoter-luciferase reporter (PAI-1 is a classic target of canonical TGF-β signaling), and observed significantly increased levels of luciferase (i.e., active TGF-β) in *Schistosoma*-exposed mice. On the other hand, we observed a decrease in active TGF-β when these mice were treated with LSKL peptide, reinforcing that activation of TGF-β by TSP-1 is critical for *Schistosoma*-PH (28).

We find similarities with other inflammation-driven etiologies of PAH, as well. TSP-1 has been reported in the plasma and skin of patients with scleroderma, a connective tissue disease that often progresses into PAH. In order to address if our findings correlate with PAH in humans, we used a bank of previously collected plasma of patients with scleroderma, drawn before and after the development of PAH. In these samples, we found the concentration of TSP-1 increased significantly after the patients developed PAH (28). TSP-1 gene expression in peripheral blood mononuclear cells in patients with scleroderma has also been reported to predict the diagnosis of PAH (30). These results are corroborated by another recent report that higher TSP-1 levels in patients with varied forms of PAH correlate with worse prognosis (31). Overall, TSP-1 likely plays a key pathological role in *Schistosoma*-PH and potentially other forms of PAH.

## Conclusion

Current therapies cannot reverse established *Schistosoma*-induced PAH in patients, and treatment options are limited to primarily vasodilator therapy. Recent investigations by others and ourselves have identified several components of the axis between Th2 inflammation, TGF-β signaling and pulmonary vascular disease, which links *Schistosoma* exposure to the subsequent development of PAH. To bring a therapy to patients, an important consideration will be to avoid immunosuppressing the individuals, who are at risk for recurrent infections due to the environment in which they live. The ideal drug target will be a downstream mediator (such as TSP-1) which is relevant in the lung pathology but is not critical to the host immune defense (such as blocking Type 2 inflammation more broadly). More studies need to be done in order to better understand the host-parasite pathophysiological interactions and identify candidate targets.

## Author Contributions

CM drafted the manuscript. BG edited the manuscript. Both CM and BG approved the final version.

### Conflict of Interest Statement

The authors declare that the research was conducted in the absence of any commercial or financial relationships that could be construed as a potential conflict of interest.
